# The challenge of performing mastoidectomy using the operating microscope with Covid-19 personal protective equipment (PPE)

**DOI:** 10.1017/S0022215120001607

**Published:** 2020-07-28

**Authors:** P J Clamp, S J Broomfield

**Affiliations:** Department of ENT Surgery, University Hospitals Bristol and Weston NHS Foundation Trust, St Michael's Hospital, Bristol, UK.

**Keywords:** COVID-19, Coronavirus, Personal Protective Equipment, Otolaryngology, Neurotology, Microsurgery, Mastoidectomy, Aerosols

## Abstract

**Objective:**

Mastoidectomy is considered an aerosol-generating procedure. This study examined the effect of wearing personal protective equipment on the view achieved using the operating microscope.

**Methods:**

ENT surgeons assessed the area of a calibrated target visible through an operating microscope whilst wearing a range of personal protective equipment, with prescription glasses when required. The distance between the surgeon's eye and the microscope was measured in each personal protective equipment condition.

**Results:**

Eleven surgeons participated. The distance from the eye to the microscope inversely correlated with the diameter and area visible (*p* < 0.001). The median area visible while wearing the filtering facepiece code 3 mask and full-face visor was 4 per cent (range, 4–16 per cent).

**Conclusion:**

The full-face visor is incompatible with the operating microscope. Solutions offering adequate eye protection for aerosol-generating procedures that require the microscope, including mastoidectomy, are urgently needed. Low-profile safety goggles should have a working distance of less than 20 mm and be compatible with prescription lenses.

## Introduction

The severe acute respiratory syndrome coronavirus-2 (SARS-CoV-2)/coronavirus disease 2019 (Covid-19) pandemic presents several previously unconsidered challenges for otological surgeons, including how to minimise the risk of coronavirus transmission to the surgeon and operating theatre staff during aerosol-generating mastoid surgery.

The mastoid air-cell system is lined with respiratory mucosa, and is continuous with the middle ear and nasopharynx via the Eustachian tube. The middle ear has been demonstrated to harbour pathogens including coronavirus.^1^ Mastoidectomy, which utilises high-speed drills within the mastoid air cells, is therefore considered to be an aerosol-generating procedure; the plume of potentially virus-containing aerosol generated by the drill may pose a risk to the surgeon and operating theatre staff.^2–4^

The UK government and specialist healthcare bodies currently recommend the use of personal protective equipment (PPE) for all surgery involving the use of high-speed drills.^5,6^ This includes, as a minimum, fluid-resistant long-sleeved gowns, gloves, filtering facepiece code 3 (FFP3) respirator masks, and eye and face protection. Whilst some degree of eye protection is conferred by surgical masks with integrated visors or by polycarbonate safety spectacles, current Public Health England guidance specifically recommends a full-face shield or visor for aerosol-generating procedures, to fully protect the eyes from potentially hazardous droplets.^5^

The challenge specific to the otological surgeon is the need to use an operating microscope when performing mastoidectomy. The full-face shield recommended for aerosol-generating procedures introduces a physical barrier, increasing the distance between the surgeon's eyes and the operating microscope eyepieces. This may reduce the microscopic view of the surgical field, making surgery more difficult and potentially increasing the risk of surgical error.

In order to quantify this concern, this study examined the effect of different forms of PPE on the operator–microscope distance and the surgical view obtained by the operator.

## Materials and methods

This study did not involve clinical care, active interventions, patients or members of the public. As such, formal ethic approval was not required. All surgeons participating in the study were consenting volunteers.

**Fig. 1.**
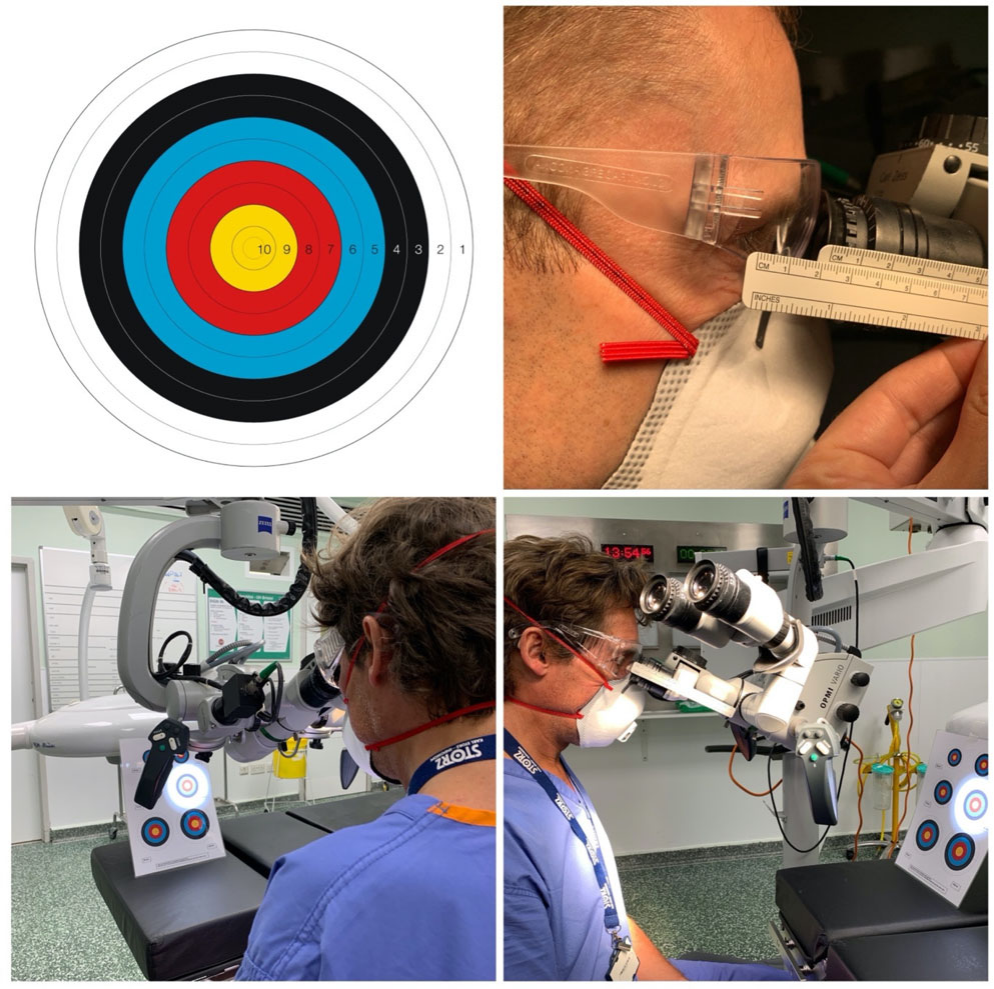
Target and study set-up, demonstrating surgeon's position and eye–microscope distance measurement.

This study utilised a Zeiss OPMI Vario S88 microscope (Carl Zeiss, Jena, Germany) in the normal position for ear surgery, which was focused on a scaled archery target with 10 equally spaced concentric circles across 5 coloured zones (Figure 1). The microscope was set with a focal length of 300 mm, and the position of the microscope and zoom were adjusted so that the target filled the entirety of the microscopic view (with the outer edge of the target matching the perimeter of the surgical view).

ENT surgeons positioned themselves for using the microscope, adopting a comfortable posture appropriate for an extended period of operating (Figure 1). Each surgeon adjusted the inter-pupillary distance, chair height and microscope angle to ensure an optimal and comfortable view.

Surgeons stated how much of the target they could see in their peripheral vision, whilst staring at the centre point with their head in the usual neutral position. A score of 1 (outer white ring) represented a full view, with progressively higher scores indicating a more restricted view visualising only a central portion of the target. The percentage of target visible was calculated from these scores (diameter and area). The distance between the most anterior aspect of the surgeon's cornea and the edge of the microscope eyepiece was measured to the nearest millimetre using a ruler secured to the side of the microscope, taking care to avoid parallax error (Figure 1).

**Fig. 2.**
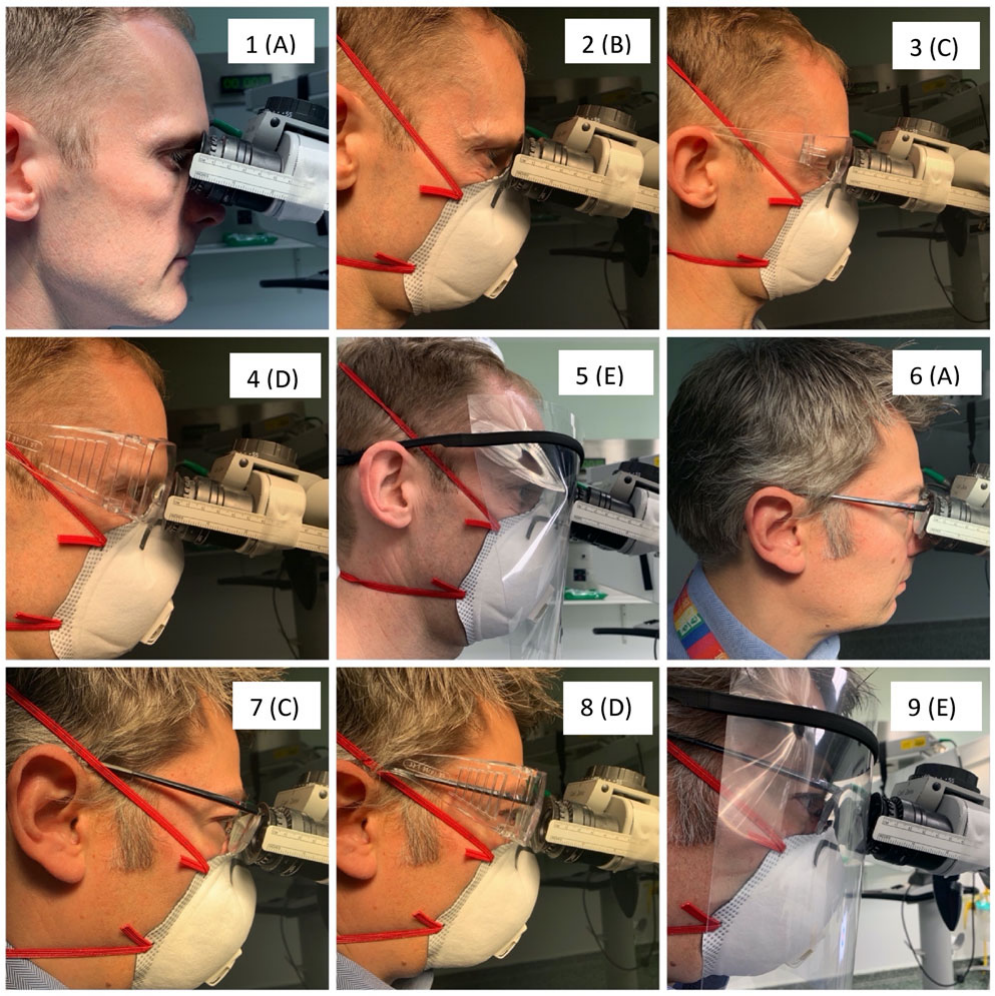
Personal protective equipment (PPE) conditions tested in surgeons with and without prescription glasses. Parts (1–5 for non-glasses wearer and 6–9 for glasses wearer) and value in parenthesis (A to E) that relates the images to PPE conditions defined in Table 1.

This process was repeated with the surgeon wearing an FFP3 mask, and various forms of eye and face protection. Scores for target view (outer-most complete ring visible) and eye–microscope distance were recorded for each PPE condition. Combinations of PPE were categorised from A to E, with A representing no PPE and E representing a full-face visor (Table 1 and Figure 2). Surgeons requiring prescription glasses for surgery continued to wear them with the additional PPE.

**Table 1.** Levels of PPE tested, including modifications for prescription spectacle wearers

PPE level	For surgeons without spectacles	For surgeons with spectacles
A. No PPE	No mask or eye protection	No mask, normal spectacles
B. FFP3 mask only	FFP3* mask, no eye protection or glasses	Not measured as surgeon requires spectacles
C. FFP3 + slim glasses	FFP3* mask, slim-line safety glasses^†^	FFP3* mask, normal spectacles
D. FFP3 + safety glasses	FFP3* mask, safety over-glasses^‡^	FFP3* mask, normal spectacles, safety over-glasses^‡^
E. FFP3 + visor	FFP3* mask, normal glasses, full-face visor	FFP3* mask, normal spectacles, full-face visor

*Model 8833 valved filtering facepiece code 3 disposable respirator (3M, Saint Paul, Minnesota, USA); conforms to European standard EN149:2001. ^†^Click Traders Ancona Clear Safety Spectacle (Beeswift, West Bromwich, UK); conforms to European standard EN166:2001. ^‡^Model PW30 Visitor Safety Spectacle (Portwest, Westport, Ireland); conforms to European standard EN166. PPE = personal protective equipment; FFP3 = filtering facepiece code 3

## Results

Eleven ENT surgeons took part, all of whom were experienced in using the operating microscope. The grade of surgeon ranged from year three specialty trainee registrar to senior consultant. Inter-pupillary distance ranged from 58 mm to 75 mm (median, 64 mm). Four of the 11 surgeons wore prescription glasses for operating, and as such could not be tested at PPE level B (FFP3 mask with no eye protection or glasses). A total of 51 measures of eye–microscope distance and target scores were collected, covering all of the defined PPE categories (A to E).

### Eye–microscope distance and target visibility

**Fig. 3.**
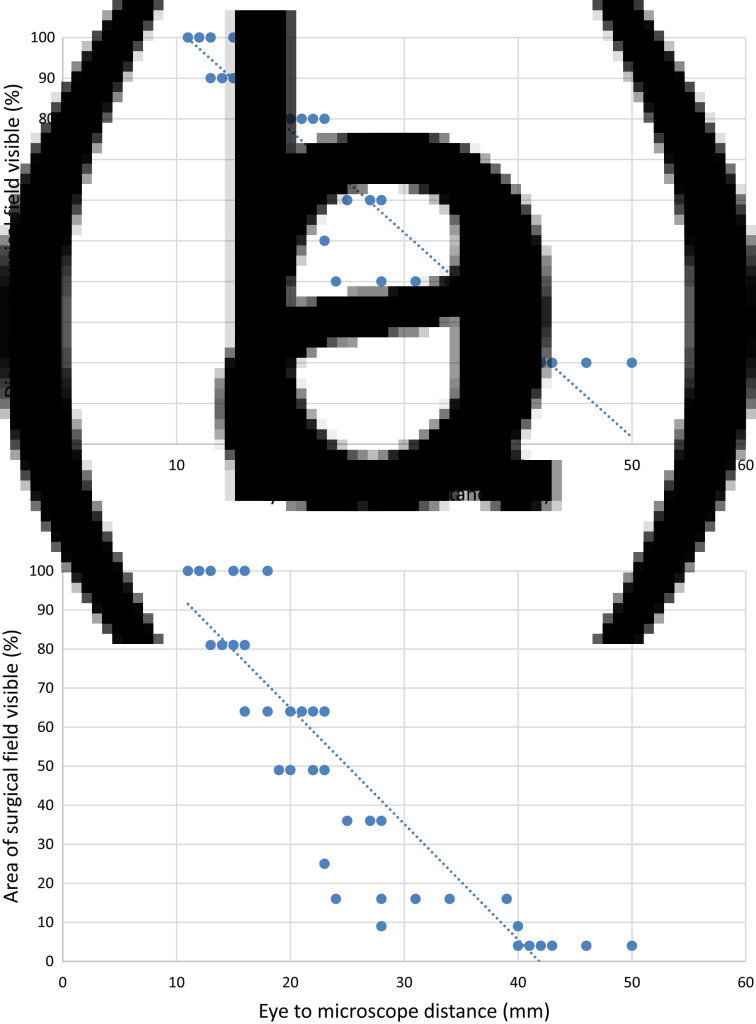
Scatter plots of results of eye–microscope measurements versus calculations of diameter (a) and area (b) of target visible.

The diameter and area of the visible target were calculated for each surgeon-reported visibility score and expressed as percentages of the overall target. The results are demonstrated in Figure 3. Both diameter and area of the target correlated highly to the eye–microscope distance (Pearson correlation co-efficient, −0.983 and −0.894 respectively; *p* < 0.001).

### Personal protective equipment effect

**Fig. 4.**
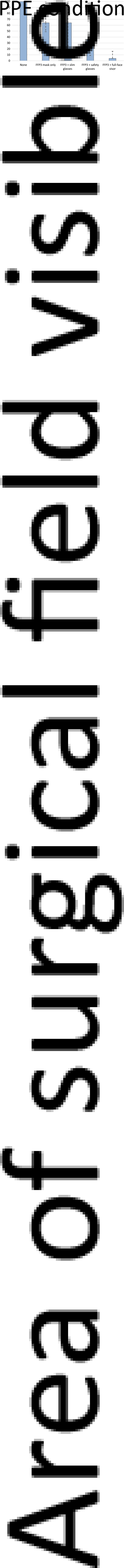
Area of target visible with each personal protective equipment (PPE) condition (median values shown with minimum–maximum range bars). FFP3 = filtering facepiece code 3

The effect of PPE on eye–microscope distance and target visibility was assessed. The median and range of eye–microscope measurements and target visibility calculations are shown in Table 2 and Figure 4. The normal eye–microscope working distance, without the use of PPE (PPE level A), was between 11 mm and 18 mm (median, 13 mm). All surgeons were able to achieve a full (100 per cent) view of the surgical field or target when not wearing any PPE (PPE level A). The use of an FFP3 mask, with progressively bulky eye protection, increased the working distance, with a resultant reduction in surgical view. The use of a full-face visor (PPE level E) reduced the area of the surgical view to a median of 4 per cent (range, 4–16 per cent).

**Table 2.** Effect of different PPE on eye–microscope distance and target visibility

PPE level	Number of measurements	Eye–microscope distance (median (range); mm)	% of target diameter visible (median (range))	% of target area visible (median (range))
A. No PPE	11	13 (11–18)	100	100
B. FFP3 mask only	7	18 (13–22)	80 (70–100)	64 (49–100)
C. FFP3 + slim glasses	11	20 (13–25)	80 (50–100)	64 (25–100)
D. FFP3 + safety glasses	11	24 (18–34)	60 (30–80)	36 (9–64)
E. FFP3 + visor	11	41 (31–50)	20 (20–40)	4 (4–16)

PPE = personal protective equipment; FFP3 = filtering facepiece code 3

## Discussion

This workplace study of practising ENT surgeons supports and quantifies our concern that using PPE reduces the view of the surgical microscopic operating field, with an inverse correlation between eye–microscope distance and target view.

The use of a full-face visor is required to satisfy the level of PPE recommended for aerosol-generating procedures such as mastoid drilling.^5^ However, the reduction in surgical view when wearing the visor in this study was severe, with less than 10 per cent of the microscopic view visible in most cases. This makes the recommended aerosol-generating procedure PPE incompatible with the use of an operating microscope at this time.

The use of FFP3 masks with the surgeon's own prescription glasses and slim-line polycarbonate safety glasses (PPE level C) or over-glasses (PPE level D) produced a highly variable effect on view, depending on the fit of the PPE to the surgeon's face and resultant increase in eye–microscope distance. Importantly, neither of these PPE levels provides the droplet protection recommended by current UK Covid-19 PPE guidelines, as the glasses do not fully protect the eyes from droplet ingress.

This study highlights the challenges facing otological surgeons in the Covid-19 era. Urgent action is needed to address the need to provide a safe operating environment for otological surgeons, whilst preserving the ability to deliver effective, safe surgical care. A number of adaptations have been suggested in order to address this risk. These include variations in surgical technique, which may obviate the need for simultaneous high-speed mastoid drilling and an operating microscope (including the use of endoscopic ear surgery, surgical loupes or three-dimensional exoscopes). Novel draping techniques, such as the ‘double-drape’ system, have also been suggested in order to reduce droplet spray.^2,7,8^ In addition, bespoke surgical shields that attach to the microscope may afford some protection to the surgeon.

Sealed, low-profile goggles could potentially provide adequate eye protection with a minimal increase in eye–microscope distance. Based on the data obtained in this study, a maximum working distance of around 20 mm is likely to preserve a view of 80 per cent diameter and 65 per cent area of the microscopic field, with an ideal working distance of 15 mm or less preserving an almost complete view of the surgical field (90 per cent diameter, 80 per cent area or more). Any such goggles should ensure complete eye protection against droplets.

Specific legislation and testing criteria are defined by the European standard EN166:2001 and American National Standards Institute Z87.1 2020. In both systems, safety glasses or goggles are tested against splash, droplet, dust and aerosol protection, with results ranging from no protection to level 5 protection. Level 4 protection corresponds to protection against fine dust and droplets over 5 μm; the authors suggest this is the minimum level of protection required for mastoidectomy. Such goggles must also be able to accommodate prescription lenses, required by around one-third of surgeons in this study.

Any modification or device designed to shield the otological surgeon's eyes and face from the aerosol generated by mastoid drilling must fulfil strict PPE requirements (preventing droplet contact with the surgeon's eyes), whilst also allowing relatively unhindered access to the microscope eyepieces. Individual surgeons may need to judge their level of confidence in performing safe otological surgery with even minor reductions in view. The data obtained in this study may be specific to the microscope and PPE used in our institute, and should be interpreted with this in mind.

## Conclusion

This study demonstrates that PPE including an FFP3 mask and full-face visor is incompatible with the use of an operating microscope; the view of the surgical field was reduced to below 10 per cent in most cases. Urgent consideration needs to be given to solutions that allow for concurrent use of the operating microscope and drill, which are required for mastoid surgery, whilst affording the surgeon adequate protection from viral transmission in the Covid-19 era. Low-profile safety goggles provide one possible solution, but a working distance of 20 mm or less should be achieved, with fully PPE-compliant sealed frames and the ability to include prescription lenses.
